# Cause-Specific Mortality in Patients Hospitalized for Myocarditis from 2004 to 2021: A Retrospective Statewide Population-Linkage Study

**DOI:** 10.3390/jcm14124089

**Published:** 2025-06-10

**Authors:** Timothy N. Kwan, Jayant Ravindran, Noor Alsadat, Gemma Kwan, David Brieger, Vincent Chow, Leonard Kritharides, Austin Chin Chwan Ng

**Affiliations:** Department of Cardiology, Concord General Repatriation Hospital, The University of Sydney, Hospital Road, Concord, NSW 2139, Australiachin.ng@sydney.edu.au (A.C.C.N.)

**Keywords:** myocarditis, heart failure, mortality, prognosis, epidemiology

## Abstract

**Background:** Myocarditis is a life-threatening condition with an increasing incidence in the past two decades. Little is known about the frequency of specific causes of death following myocarditis. This study aimed to identify the different causes of death after myocarditis diagnosis and determine factors associated with mortality. **Methods:** We conducted a retrospective population-wide observational study in New South Wales (NSW), Australia from July 2004 to September 2021. Data were attained from the NSW Admitted Patient Data Collection database and death was tracked from the death registry to 31 March 2022. Cause of death was ascertained from manual reviews of all death certificates and adjudicated independently by three reviewers. **Results:** Among 4071 unique index admissions for myocarditis (median age: 42 years; 66% male), cumulative all-cause mortality was 4.5% in-hospital, 8.2% at 1 year, 13.3% at 5 years and 15.5% by the end of follow up (median 5.3 years). Within 30 days of admission, the leading cause of death was cardiovascular (66%), including myocarditis (36%) and heart failure (12%). Non cardiovascular causes accounted for 32% of deaths and included infection (17%) and malignancy (6%). Beyond 30 days, cardiovascular deaths declined to 34% (only 3% due to myocarditis). Higher mortality risk was associated with older age, higher Charlson comorbidity index, and myocarditis complicated by intensive care unit admission, heart failure, stroke, or arrhythmia. **Conclusions:** Patients admitted with myocarditis face significant mortality risks. The highest mortality occurs within the first 30 days, predominantly due to cardiovascular causes, although after 30 days the predominant cause of death shifts to non-cardiovascular causes.

## 1. Introduction

Myocarditis is a complex and heterogeneous disease with poorly understood long-term sequelae. Traditionally, myocarditis has been recognized as a potentially life-threatening condition in which patients are at risk of cardiogenic shock and lethal arrhythmias. In-hospital mortality approached 50% in reported case series of fulminant myocarditis [1]. However, advances in diagnostic techniques, including high-sensitivity troponin assays and cardiac magnetic resonance imaging, have led to the identification of more subclinical and asymptomatic cases. Recently an unselected myocarditis cohort reported in-hospital mortality of 3.2% [2,3].

Myocarditis can present in various forms including acute, chronic active and chronic persistent myocarditis [4], each with distinct implications for long-term outcomes. Even after the resolution of inflammation, myocarditis can lead to adverse remodeling, left ventricular dysfunction and myocardial fibrosis [5]. These pathological changes contribute to the development of chronic cardiomyopathies, often necessitating heart failure therapies to promote reverse remodeling [6]. Additionally, myocarditis is associated with an increased risk of thromboembolic events, particularly in the presence of left ventricular dysfunction or atrial fibrillation, necessitating anticoagulation considerations [7]. Some patients may also require arrhythmia management, either through pharmacotherapy or implantable cardioverter-defibrillator (ICD) placement [8,9].

Various features of myocarditis have been recognized as relevant to prognostication. The most well recognized prognostic factor for mortality after myocarditis is age [10]. Myocarditis can further be risk stratified based on clinical variables such as left ventricular dysfunction and arrhythmias [11]. Since 2020, a significant proportion of myocarditis cases have been associated with COVID-19, with incidence rates varying between 10% and 50%, depending on the population studied [12,13]. There is evidence COVID-19 myocarditis carries a mortality risk approximately five times higher than myocarditis from other causes [12].

While the immediate and long-term cardiac consequences of myocarditis are increasingly recognized, the broader impact on non-cardiac health remains unclear. There is a lack of long-term mortality data on myocarditis with previous longitudinal studies not describing the breadth of cardiovascular and noncardiovascular causes of death [3,14]. No studies have systematically reported cause-specific mortality trends in a myocarditis cohort, the incidence of non-cardiac illnesses or assessed whether these patterns have changed in recent years.

This study aims to address this gap by examining long-term, cause-specific mortality in an unselected statewide myocarditis cohort over an 18-year period.

## 2. Materials and Methods

This population-wide retrospective cohort study included all hospital admissions for myocarditis in New South Wales (NSW), the most populous state in Australia, between 1 July 2001, and 31 March 2022. Data were extracted from the NSW Admitted Patient Data Collection (APDC) database, which captures over 97% of healthcare facility admission records in NSW. Myocarditis cases were identified using International Statistical Classification of Diseases and Related Health Problems, Tenth Revision, Australian Modification (ICD-10-AM) codes (Supplementary Table S1), included as either a primary or secondary diagnosis.

To ascertain mortality outcomes, data were linked to the NSW death registry through the Centre for Health Record Linkage (CHeReL). Death records were available until 31 March 2022, marking the end of the study follow-up period.

Patients were excluded if they were (1) admitted with myocarditis before 1 July 2004 to ensure a minimum three-year look-back period for background data, (2) had their first episode of myocarditis after 30 September 2021 to ensure at least a 6-month follow-up or (3) not a New South Wales resident to minimize loss to follow up (Supplementary Figure S1).

Two reviewers (J.R. and N.A.) independently classified the cause of death from free text entries from death certificates into cardiovascular and non-cardiovascular categories. Cardiovascular causes included myocarditis, heart failure, myocardial infarction, arrhythmia, stroke and pulmonary embolism. Noncardiovascular causes included infection, malignancy, pulmonary disease, other non-cardiac death. Cases with insufficient data were classified as unknown cause of death. This classification was adapted from prior literature [15]. Each patient was assigned a single primary cause of death. Where there was a disagreement, the cause of death was adjudicated by a third reviewer (T.K.) and the final classification decided by consensus. All reviews of death certificates were blinded to patient’s background history.

Patient characteristics were analyzed with respect to their index myocarditis admission. Background conditions were identified using ICD-10-AM codes recorded during both the index hospitalization and any prior admissions. Myocarditis complications, including atrial fibrillation, ventricular arrhythmias, heart failure, and stroke, were defined as new diagnoses recorded during the index hospitalization in patients without prior history of these conditions. In-hospital mortality was defined as death occurring during the index hospitalization, before discharge. Time to death was measured from the date of admission for myocarditis to the recorded date of death.

Survival after myocarditis admission was estimated with Kaplan–Meier plots, with three pre-specified stratified subgroups of interest defined according to intensive care unit (ICU) admission, median age and COVID-19 myocarditis. COVID-19 myocarditis was defined as myocarditis within 30 days of an admission for COVID-19 [13,14]. As COVID-19 myocarditis was only present after the onset of the COVID-19 pandemic in Australia, the analysis of COVID-19 myocarditis was restricted to after 25 January 2020. COVID-19 myocarditis was specifically infection-related and not based on the recency of vaccination.

The cause-specific death rates were counted based on (1) date of admission stratified into quartiles (separated by the dates 3 July 2004, 22 April 2011, 12 November 2015, 26 December 2018 and 28 September 2021), and (2) the time from index myocarditis admission to death (<30 days, 30–365 days and >365 days). Poisson regression was used to evaluate whether the distribution of causes of death changed over time, treating time since myocarditis as an ordinal variable of three possible values. Cumulative incidence functions were plotted using the Fine and Gray method to account for competing risks.

Factors associated with long-term mortality following myocarditis admission were analyzed using univariate and multivariate Cox proportional hazards models. Pre-specified variables considered for this analysis were age, sex, ICU admission, remoteness of hospital as measured by the Australian Statistical Geography Standard [16], background of chronic kidney disease, diabetes, ischemic heart disease, hypertension, hyperlipidemia, chronic obstructive pulmonary disease, liver disease, autoimmune disease, malignancy, COVID-19 myocarditis, years since beginning of the study period and myocarditis admission complicated by atrial fibrillation, heart failure, ventricular arrhythmia and stroke. The predictive power of each Cox regression model was measured by Harrell’s C-index.

A significance threshold of *p* < 0.05 was applied for all statistical tests. Analyses were conducted using R version 4.3.1. Ethics approval was granted by NSW Population and Health Services Research Ethics Committee, reference number: 2019/ETH01790, who also granted a waiver of the usual requirement for the consent of the individual to the use of their health information. The study adhered to the principles outlined in the 2024 Declaration of Helsinki.

## 3. Results

Among 4071 patients diagnosed with myocarditis, the median age was 42 years, with two-thirds male. ICU admission was required in 15% of cases, 27% had a background of ischemic heart disease, 24% heart failure, 13% atrial fibrillation, 8% malignancy and 6% had autoimmune disease. Myocarditis admissions were complicated by heart failure (14.3%), atrial fibrillation (5.9%), ventricular arrhythmia (4.0%) and stroke (0.7%) (Table 1). Autopsy results were not available, however, of the patients who died in hospital (n = 182, 4.5%) only 41 patients had autopsies. This population has previously been described [13].

**Figure 1:**
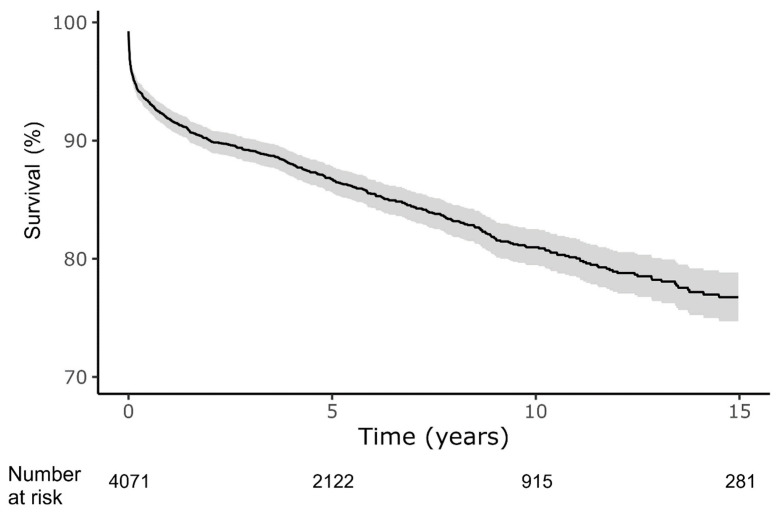
Survival after index myocarditis diagnosis. Shaded area represents 95% confidence interval. Analysis according to the Kaplan–Meier method.

In-hospital mortality occurred in 4.5% of patients, with cumulative 30-day, 1-year, 5-year and end-of-study (median follow-up 5.3 years) crude mortality of 3.4%, 8.2%, 13.3% and 15.5%, respectively (Figure 1). The initial mortality rate was high at 3.4% within the first month but after a year the mortality rate stabilized to 1.0% per year. Patients who required ICU admission during their hospitalization were significantly more likely to die during the first month after admission, however, during the remaining 15 years of follow-up had approximately the same death rate as their counterparts who were not admitted to ICU (Supplementary Figure S2). Older patients (>42 years) had higher in-hospital and long-term mortality (Supplementary Figure S3).

**Figure 2:**
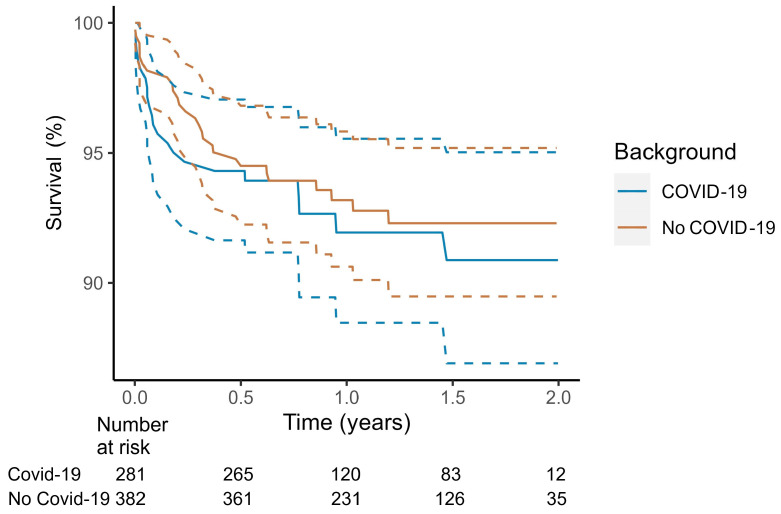
Survival after index myocarditis diagnosis stratified by COVID-19. COVID-19 associated myocarditis if myocarditis occurred within 30 days of index myocarditis hospitalization. Dotted lines demarcate 95% confidence interval. Analysis according to the Kaplan–Meier method, from the onset of the COVID-19 pandemic in Australia 25 January 2020.

Following the first confirmed COVID-19 case in Australia (25 January 2020), myocarditis-related mortality remained similar to other types of myocarditis according to Kaplan–Meier analysis (Figure 2). After COVID-19 myocarditis admission, 30-day mortality was 3.5%, and 1-year mortality was 8.0%.

Overall, more patients died from noncardiovascular causes of death (54%) than cardiovascular causes of death (43%) over the entire study period. Only 12% of patients were listed as dying from myocarditis according to their death certificate. Other cardiovascular deaths not reported as being related to myocarditis include the following: 16% died from heart failure, 13% from myocardial infarction, 3% from arrhythmia and 3% from stroke (Table 2). There were no significant differences between the sexes for causes of death.

The distribution of cause-specific mortality shifted over time with cardiovascular mortality predominating early (<30 days, 66%) and noncardiovascular mortality the predominating later (>30 days, 62%). In particular almost all (63/78; 81%) of deaths attributable to myocarditis occurred within a month of myocarditis hospitalization (Table 3).

Despite the young age of patients in the current study, death from malignancy was relatively common and accounted for 22% of deaths beyond 30 days after myocarditis diagnosis. Patients who died from malignancy were not older than patients who died from other causes (median age 65.1 vs. 66.5 years). In patients who died from malignancy, 40 (40%) did not have previous or current malignancy at the time of myocarditis diagnosis.

Between 2004 and 2021, the proportion of cardiovascular deaths at 6-month follow-up declined, with a corresponding rise in noncardiovascular deaths particularly from malignancy (Table 4). During the study period it became much less common for myocarditis patients to die from myocardial infarction. Similar trends were also seen for in-hospital and 30-day cause-specific mortality over time (Supplementary Table S2 and Supplementary Table S3, respectively).

As with patients in the cohort from 2018 to 2021, COVID-19 myocarditis patients died mostly (62%) from non-cardiovascular causes (Supplementary Table S4).

**Figure 3:**
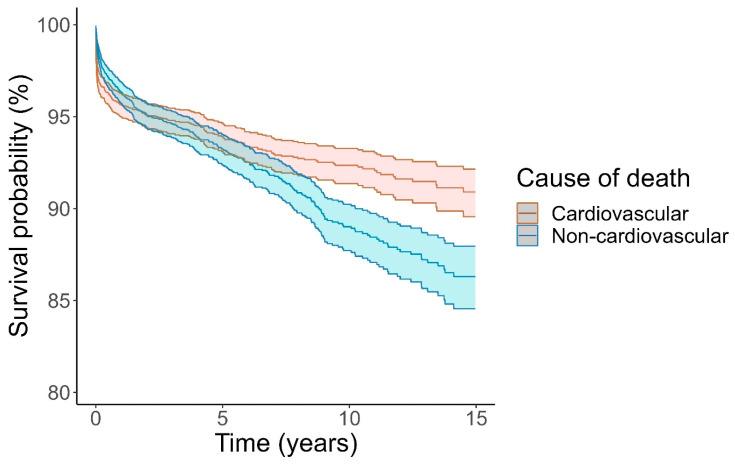
Cumulative incidence of death according to cardiovascular or noncardiovascular death. Causes of death treated as competing risks according to Fine and Gray. Unknown causes of death (n = 18) excluded. Shaded area indicates 95% confidence interval.

**Figure 4:**
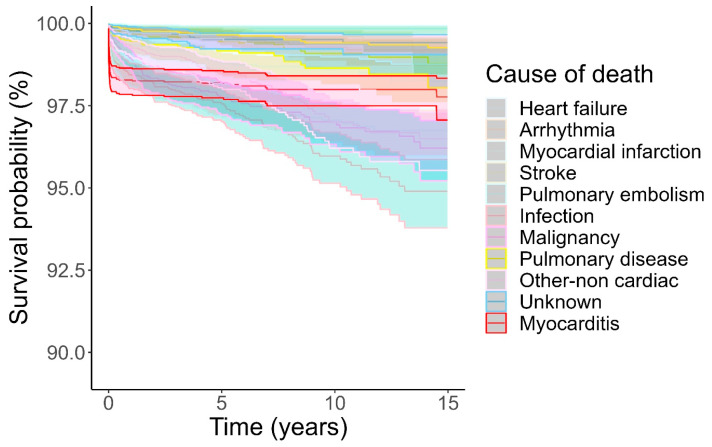
Cumulative incidence of death according to cause of death. Death due to myocarditis highlighted in red. Causes of death treated as competing risks according to Fine and Gray. Shaded area indicates 95% confidence interval.

Competing risk analysis confirmed that cardiovascular causes of death were more common early and non-cardiovascular causes of death were more common later (Figure 3). Results from Kaplan–Meier analysis (Supplementary Figure S4) were almost identical. In particular, deaths attributed directly to myocarditis occurred overwhelmingly early, during the first month from myocarditis admission, and by comparison, death from myocarditis for the next 15 years was very rare (Figure 4).

Multivariable analysis identified older age, ICU admission and specific complications (atrial fibrillation, heart failure, ventricular arrhythmia and stroke) as significant predictors of mortality (Table 5). The comorbidities malignancy, chronic kidney disease, diabetes, liver disease and chronic obstructive pulmonary disease were also independently associated with higher mortality. Notably, female sex was associated with higher unadjusted mortality (Supplementary Table S5), but this effect was negated after adjusting for comorbidities. A separate model restricted to time since the onset of the COVID-19 pandemic confirmed that COVID-19 myocarditis was not associated with higher mortality (HR 0.90, 95% CI 0.47–1.73, *p* = 0.757, Supplementary Table S6).

## 4. Discussion

In this retrospective population-wide cohort study spanning 18 years, mortality was attributable to a broad range of causes. The highest death rate was immediately after hospitalization, with a 4.5% in-hospital mortality followed by a steady 1% death rate per year during subsequent years. Early deaths within the first month were predominantly cardiovascular in origin, with more than half directly attributable to myocarditis. In contrast, beyond 30 days, mortality was primarily due to noncardiovascular causes, with infections and malignancies accounting for two-thirds of these deaths. Myocarditis patients had a higher mortality rate if they were older, had more comorbidities or experienced complications such as ICU admission, heart failure, stroke and arrhythmia. Sex was not predictive of mortality after adjustment.

The present findings suggest that mortality attributable to myocarditis or its direct complications is mostly an early event. Myocarditis was the attributed cause of death in 37% of cases within 30 days but only 3% of cases beyond this period. Although chronic and recurrent myocarditis are well described, they seem to rarely contribute to delayed deaths. This is consistent with our previous research demonstrating that repeat hospitalizations for myocarditis even as a non-primary diagnosis are very rare [13]. By contrast, the shift in cause-specific mortality after 30 days highlights the high burden of comorbidities in this population, especially malignancy and infection, that drive long-term outcomes. We speculate that some malignancies may have been an extension of the pathological process that initially triggered myocarditis and some infections could reflect lingering immune dysregulation or immunosuppression [17].

A notable finding was the high prevalence of malignancy in this myocarditis cohort (8%) especially in the patients who died (25%), despite their young age. This corresponds with the well-recognized etiologies of myocarditis that include cancer and cancer therapy [18]. Strikingly, 40% of patients who succumbed to malignancy had no prior cancer diagnosis at the time of their myocarditis presentation, suggesting that myocarditis may, in some cases, be an early manifestation of undiagnosed malignancy. Myocarditis may occur as a paraneoplastic phenomenon, as reported in several case reports [19]. Even in the absence of specific pathogenic antibodies, it can arise secondary to the hyperinflammatory state associated with cancer, which in its most severe form, may manifest as cytokine storm [20]. This particular study does not explain the reason for this association and it should be acknowledged that there are alternative explanations for an association such as (a) patients with myocarditis and malignancy share certain risk factors or that myocarditis such as systemic autoimmune diseases [21] or genetics [22], (b) a chronic inflammatory milieu perhaps due to the myocarditis can cause malignancy [23] and (c) patients who present for medical attention for myocarditis may be more likely to be screened for other diseases such as cancer. Further research exploring the relationship between myocarditis and malignancy is required.

### 4.1. Comparison with Existing Literature

This is the first study to assess the varied causes of death after myocarditis. While previous studies have examined long-term outcomes in myocarditis patients, cause-specific mortality has not been well characterized. The overall in-hospital mortality rate observed in our study is within the range of previously reported rates of 2.5% [2], 3.2% [3] or 5.3% [24]. The survival curve, with the highest mortality occurring within the first 30 days, is also consistent with prior findings [10,25]. Previous research has noted that cardiovascular deaths account for approximately half [25] or “most” [26] deaths after myocarditis. However, to our knowledge, no prior study has provided a detailed breakdown of mortality causes following myocarditis or shown how they have changed over time.

Several prognostic factors identified in this study align with existing literature. It is known, and perhaps unsurprising, that older adults have higher mortality after myocarditis both immediately and during a decade of follow-up [10,25]. The present study also aligns with previous research that sex is not predictive of mortality [27,28], despite it being relevant in non-ischemic cardiomyopathy more broadly [29].

The impact of COVID-19-related myocarditis has been a topic of recent investigation. Contrary to prior studies suggesting a worse prognosis for COVID-19 myocarditis [14], our findings did not support this association. We hypothesize that evolving diagnostic criteria may account for this discrepancy. In the recent French study, COVID-19 myocarditis comprised only 6% of myocarditis cases [14], whereas in the present Australian cohort, myocarditis was diagnosed much more commonly after COVID-19, with COVID-19 myocarditis accounting for nearly 50% of contemporary cases [13].

Prognostic implications of other clinical features such as ICU admission, cardiovascular complications, and comorbidities remain underexplored in the literature. Our findings indicate that these factors are significantly associated with increased mortality, which is clinically intuitive. Additionally, while previous studies have correlated reduced systolic function and late gadolinium enhancement with worse outcomes in myocarditis [27,30], this is outside the scope of the present study in which imaging data were unavailable.

### 4.2. Strengths and Limitations

A major strength of this study is its large sample size and long-term follow-up over 18 years, which enabled statistically robust and novel insights into myocarditis outcomes. However, several limitations must be acknowledged. The study cohort was identified from the NSW Admission-Patient-Data-Collection database, which, despite containing over 97% of all NSW hospital admissions, lacks granular clinical details, such as medication use, laboratory results, and imaging findings. The severity of myocarditis had to be inferred from indicators like ICU admission. The index myocarditis admissions were indirectly identified using a three-year look-back strategy to exclude prior episodes, rather than explicit documentation.

Due to the administrative nature of the APDC database, the diagnoses for myocarditis and all other diagnoses relied on the diagnosis coded by clinicians and could not be independently verified. Nevertheless, ICD-10 codes have previously been shown to be robust in Australia [31], and the accuracy of myocarditis specific ICD-10 codes have been shown to be robust outside of Australia [25,32]. The classifications of myocarditis, such as COVID-19 myocarditis, was only inferred from associations. Additionally, the acuity of ICD-10 codes was not explicitly coded so had to be deduced from the pattern of admission; for example, acute diagnoses or complications were defined as such at the first recorded episode.

Finally, the cause of death data were derived from death certificates and detailed clinical data or autopsy findings were not available. This is an important limitation as previous autopsy studies suggest that myocarditis is frequently underdiagnosed [33,34]. In general, death certificates often misclassify cause of death when judged by autopsy findings [35]. Myocarditis can be a difficult diagnosis, which is often revised after further review; for example, in one study, more than half of patients with suspected myocarditis at presentation were reclassified into other cardiomyopathies after performance of cardiac MRI [36]. Furthermore, autopsies were only performed on a small minority of patients in the present study in keeping with the known reduction in autopsy rates internationally [37]. Although the cause of death had been decided by clinicians involved in the patients’ clinical care, it could only be broadly defined and was not always mutually exclusive; for example, deaths attributable to myocarditis overlapped with those due to arrhythmia and heart failure. It is likely some cases of death from heart failure and arrhythmia had significant contributions from either acute or chronic myocarditis that was not recognized or documented on the death certificate. Although this was a subjective process, the causes of death were defined according to best practice by the consensus of expert clinicians. The present study reflects clinically recognized diagnoses rather than subclinical or undiagnosed myocarditis cases.

## 5. Conclusions

This study provides important insights into the causes of death following myocarditis. After the acute phase, death rates were low, especially death directly attributable to myocarditis. While cardiovascular mortality predominates within the first 30 days, long-term mortality is driven by noncardiovascular causes, particularly infections and malignancies. These findings underscore the importance of holistic long-term follow up for these patients. Older age, comorbidities and a complicated myocarditis admission were all negative prognostic markers. Future research incorporating imaging and biomarker data could further refine risk stratification and improve management strategies for this patient population.

## Figures and Tables

**Table 1:** Summary features of patients admitted at time of index case of myocarditis.

Feature	All Patients (n = 4071)	Patients Who Died (n = 632)	*p*-Value ^a^
Sex (male)	66.1% (2690/4071)	57.9% (366/632)	<0.001
Age, years	42.4 (27.2–58.7)	65.1 (49.2–77.3)	<0.001
Intensive care unit admission	15.4% (625/4071)	30.4% (192/632)	<0.001
City hospital ^b^	73.9% (3007/4071)	72.8% (460/632)	0.42
Rural hospital ^b^	5.8% (235/4071)	5.2% (33/632)	0.562
In-hospital mortality	4.5% (182/4071)	100% (632/632)	-
Complicated by heart failure	14.3% (584/4071)	27.4% (173/632)	<0.001
Complicated by atrial fibrillation	5.9% (239/4071)	13.3% (84/632)	<0.001
Complicated by ventricular arrhythmia	4.0% (162/4071)	7.4% (47/632)	<0.001
Complicated by stroke	0.7% (29/4071)	2.5% (16/632)	<0.001
Background ischemic heart disease	27.3% (1110/4071)	44.9% (284/632)	<0.001
Background chronic obstructive pulmonary disease	11.9% (485/4071)	20.6% (130/632)	<0.001
Background malignancy	7.9% (321/4071)	25.2% (159/632)	<0.001
Background autoimmunity	5.6% (229/4071)	9.8% (62/632)	<0.001
Background chronic kidney disease	4.6% (187/4071)	15% (95/632)	<0.001
Background diabetes	4.6% (187/4071)	23.6% (149/632)	<0.001
Background hypertension	26.8% (1090/4071)	50.9% (322/632)	<0.001
Background hyperlipidaemia	7.9% (320/4071)	15.8% (100/632)	<0.001
Background liver disease	7.7% (315/4071)	17.2% (109/632)	<0.001
Charlson comorbidity index	1 (0–3)	5 (2.8–7)	<0.001

Continuous variables reported as median (IQR). Binary variables reported as percentage (n). ^a^. Statistical comparison was based on patients who died versus survivors during study period. ^b^. City hospital defined by Australian Statistical Geography Standard 1 and rural defined by Australian Statistical Geography Standard 3 or more.

**Table 2:** Cause of death after diagnosis of myocarditis according to sex.

Cause of Death	Proportion of All Death	Proportion of All Death in Males	Proportion of All Death in Females	*p*-Value
Cardiovascular	42.9% (271/632)	45.1% (165/366)	39.8% (106/266)	0.218
Heart failure	16% (101/632)	17.2% (63/366)	14.3% (38/266)	0.378
Myocarditis	12.3% (78/632)	13.4% (49/366)	10.9% (29/266)	0.415
Myocardial infarction	7.9% (50/632)	8.7% (32/366)	6.8% (18/266)	0.448
Arrhythmia	3.2% (20/632)	3% (11/366)	3.4% (9/266)	0.970
Stroke	2.5% (16/632)	2.2% (8/366)	3% (8/266)	0.694
Pulmonary embolism	0.9% (6/632)	0.5% (2/366)	1.5% (4/266)	0.418
Noncardiovascular	54.3% (343/632)	52.5% (192/366)	56.8% (151/266)	0.321
Infection	20.9% (132/632)	19.9% (73/366)	22.2% (59/266)	0.560
Malignancy	16% (101/632)	16.4% (60/366)	15.4% (41/266)	0.824
Other non-cardiac death	13% (82/632)	13.1% (48/366)	12.8% (34/266)	0.998
Pulmonary disease	4.4% (28/632)	3% (11/366)	6.4% (17/266)	0.065
Unknown cause of death	2.8% (18/632)	2.5% (9/366)	3.4% (9/266)	0.654

Follow up was the entire duration of study from 1 July 2001 to 31 March 2022. Total cases of myocarditis numbered 4071. *p*-value calculated by Poisson regression, treating time since myocarditis as an ordinal variable of 3 possible values.

**Table 3:** Cause of death after diagnosis of myocarditis, stratified by time since myocarditis diagnosis.

Cause of Death	<30 Days Since Myocarditis	30–365 Days Since Myocarditis	>365 Days Since Myocarditis	*p*-Value
Cardiovascular	115 (65.7%)	60 (38.7%)	96 (31.8%)	0.004
Heart failure	21 (12%)	39 (25.2%)	41 (13.6%)	0.770
Myocarditis	63 (36%)	7 (4.5%)	8 (2.6%)	0.285
Myocardial infarction	20 (11.4%)	6 (3.9%)	24 (7.9%)	<0.001
Arrhythmia	4 (2.3%)	3 (1.9%)	13 (4.3%)	0.115
Stroke	5 (2.9%)	2 (1.3%)	9 (3%)	0.183
Pulmonary embolism	2 (1.1%)	3 (1.9%)	1 (0.3%)	1
Noncardiovascular	56 (32%)	91 (58.7%)	196 (64.9%)	0.233
Infection	30 (17.1%)	33 (21.3%)	69 (22.8%)	0.106
Malignancy	10 (5.7%)	33 (21.3%)	58 (19.2%)	0.573
Other non-cardiac death	11 (6.3%)	17 (11%)	54 (17.9%)	0.686
Pulmonary disease	5 (2.9%)	8 (5.2%)	15 (5%)	0.842
Unknown cause of death	4 (2.3%)	4 (2.6%)	10 (3.3%)	0.085
Total	175 (100%)	155 (100%)	302 (100%)	<0.001

Time since myocarditis measured from the time of index myocarditis admission. *p*-value calculated by Poisson regression, treating time since myocarditis as an ordinal variable of 3 possible values.

**Table 4:** Changing pattern of 6-month cause-specific mortality after diagnosis of myocarditis over time.

Cause of Death	Quartile 1: 2004–2011	Quartile 2: 2011–2015	Quartile 3: 2015–2018	Quartile 4: 2018–2021	*p*-Value
Cardiovascular	52 (60.5%)	47 (64.4%)	30 (58.8%)	22 (34.4%)	<0.001
Heart failure	11 (12.8%)	19 (26.0%)	11 (21.6%)	5 (7.8%)	0.089
Myocarditis	20 (23.3%)	22 (30.1%)	15 (29.4%)	13 (20.3%)	0.136
Myocardial infarction	16 (18.6%)	4 (5.5%)	0 (0%)	2 (3.1%)	<0.001
Arrhythmia	3 (3.5%)	1 (1.4%)	2 (3.9%)	0 (0%)	0.163
Stroke	0 (0%)	1 (1.4%)	2 (3.9%)	2 (3.1%)	0.183
Pulmonary embolism	2 (2.3%)	0 (0%)	0 (0%)	0 (0%)	1
Noncardiovascular	31 (36%)	24 (32.9%)	21 (41.2%)	41 (64.1%)	0.265
Infection	15 (17.4%)	15 (20.5%)	10 (19.6%)	13 (20.3%)	0.500
Malignancy	8 (9.3%)	2 (2.7%)	4 (7.8%)	17 (26.6%)	0.023
Other non-cardiac death	7 (8.1%)	3 (4.1%)	4 (7.8%)	9 (14.1%)	0.515
Pulmonary disease	1 (1.2%)	4 (5.5%)	3 (5.9%)	2 (3.1%)	0.777
Unknown cause of death	3 (3.5%)	2 (2.7%)	0 (0%)	1 (1.6%)	0.163
Total	86 (100%)	73 (100%)	51 (100%)	64 (100%)	-
Total mortality for each quartile cohort	86/1018 (8.4%)	73/1018 (7.2%)	51/1017 (5.0%)	64/1018 (6.3%)	0.018

*p*-value calculated by Poisson regression, treating time of diagnosis of myocarditis as an ordinal variable of 4 possible values. The 4 quantiles were separated by the precise dates: 3 July 2004, 22 April 2011, 12 November 2015, 26 December 2018 and 28 September 2021.

**Table 5:** Predictors of all-cause mortality following myocarditis admission in multivariable model ^a^.

Variable	n	Adjusted Hazard Ratio (95% CI)	*p*-Value
Age (per 10 years)	4071	1.52 (1.44–1.6)	<0.001
Sex (male)	2690	1.02 (0.86–1.2)	0.832
ICU	625	2.1 (1.73–2.54)	<0.001
ASGS ^b^	3007	0.97 (0.85–1.11)	0.64
Background chronic kidney disease	187	2.04 (1.61–2.6)	<0.001
Background diabetes	423	1.36 (1.1–1.67)	0.004
Background ischemic heart disease	1110	1.06 (0.89–1.27)	0.515
Background hypertension	1090	1.05 (0.86–1.28)	0.623
Background hyperlipidaemia	320	0.76 (0.6–0.97)	0.027
Background chronic obstructive pulmonary disease	485	1.71 (1.39–2.09)	<0.001
Background liver disease	315	1.86 (1.48–2.33)	<0.001
Background autoimmune disease	229	1 (0.76–1.32)	0.982
Background malignancy	321	2.57 (2.1–3.14)	<0.001
Complicated by atrial fibrillation	239	0.86 (0.67–1.1)	0.233
Complicated by heart failure	584	1.48 (1.23–1.78)	<0.001
Complicated by ventricular arrhythmia	162	1.46 (1.07–2)	0.018
Complicated by stroke	29	2.67 (1.6–4.44)	<0.001
Time since 2004 (years)	4071	0.93 (0.91–0.94)	<0.001

^a^. C-index of model: 0.82; refer to Supplementary Table S6 for univariable analysis. ^b^. ASGS: Australian Statistical Geography Standard, where higher number associated with more remoteness (i.e., hazard ratio below 1 indicates lower death in more remote centers).

## Data Availability

The original data presented in the study are available in NSW Admitted Patient Data Collection (APDC) database and the NSW Registry of Births, Deaths & Marriages. These data can be attained on application to the NSW government but are not owned by the study authors.

## Supplementary Materials

The following supporting information can be downloaded at: https://www.mdpi.com/article/10.3390/jcm14124089/s1, Supplementary Figure S1: Patient selection flowchart; Supplementary Figure S2: Kaplan–Meier plot of survival after myocarditis diagnosis stratified by admission to intensive care unit during index hospitalization; Supplementary Figure S3: Kaplan–Meier plot of survival after myocarditis diagnosis stratified by median age (42 years) at index hospitalization; Supplementary Figure S4: Kaplan–Meier plot of survival after myocarditis diagnosis according to cardiovascular and non-cardiovascular causes of death; Supplementary Table S1: The International Classification of Disease (ICD-10AM) and Australian Classification of Health Intervention (ACHI) codes used in the present study; Supplementary Table S2: Changing pattern of in-hospital cause-specific mortality after diagnosis of myocarditis over time; Supplementary Table S3: Changing pattern of 30-day cause-specific mortality after diagnosis of myocarditis over time; Supplementary Table S4: Cause-specific mortality in patients with COVID-19 myocarditis; Supplementary Table S5: Univariable predictors of mortality following myocarditis admission; Supplementary Table S6: Predictors of mortality following myocarditis admission in multivariable model in cohort restricted to after COVID-19 pandemic.

## Abbreviations

The following abbreviations are used in this manuscript:

ICD	implantable cardioverter-defibrillator
NSW	New South Wales
ICD-10-AM	International Statistical Classification of Diseases and Related Health Problems, Tenth Revision, Australian Modification
CHeReL	Centre for Health Record Linkage

## References

[1] Sharma A.N., Stultz J.R., Bellamkonda N., Amsterdam E.A. (2019). Fulminant myocarditis: Epidemiology, pathogenesis, diagnosis, and management. Am. J. Cardiol..

[2] Ozierański K., Tymińska A., Kruk M., Koń B., Skwarek A., Opolski G., Grabowski M. (2021). Occurrence, trends, management and outcomes of patients hospitalized with clinically suspected myocarditis—Ten-year perspectives from the MYO-PL nationwide database. J. Clin. Med..

[3] Ammirati E., Cipriani M., Moro C., Raineri C., Pini D., Sormani P., Mantovani R., Varrenti M., Pedrotti P., Conca C. (2018). Clinical presentation and outcome in a contemporary cohort of patients with acute myocarditis: Multicenter Lombardy registry. Circulation.

[4] Lieberman E.B., Hutchins G.M., Herskowitz A., Rose N.R., Baughman K.L. (1991). Clinicopathoiogic description of myocarditis. J. Am. Coll. Cardiol..

[5] Tschöpe C., Ammirati E., Bozkurt B., Caforio A.L., Cooper L.T., Felix S.B., Hare J.M., Heidecker B., Heymans S., Hübner N. (2021). Myocarditis and inflammatory cardiomyopathy: Current evidence and future directions. Nat. Rev. Cardiol..

[6] Peretto G., Sala S., Rizzo S., Palmisano A., Esposito A., De Cobelli F., Campochiaro C., De Luca G., Foppoli L., Dagna L. (2020). Ventricular arrhythmias in myocarditis: Characterization and relationships with myocardial inflammation. J. Am. Coll. Cardiol..

[7] Mizia-Stec K., Caforio A.L., Charron P., Gimeno J.R., Elliott P., Kaski J.P., Maggioni A.P., Tavazzi L., Rigopoulos A.G., Laroche C. (2020). Atrial fibrillation, anticoagulation management and risk of stroke in the Cardiomyopathy/Myocarditis registry of the EURObservational Research Programme of the European Society of Cardiology. ESC Heart Fail..

[8] Peretto G., Sala S., Rizzo S., De Luca G., Campochiaro C., Sartorelli S., Benedetti G., Palmisano A., Esposito A., Tresoldi M. (2019). Arrhythmias in myocarditis: State of the art. Heart Rhythm..

[9] Smith E.D., Lakdawala N.K., Papoutsidakis N., Aubert G., Mazzanti A., McCanta A.C., Agarwal P.P., Arscott P., Dellefave-Castillo L.M., Vorovich E.E. (2020). Desmoplakin cardiomyopathy, a fibrotic and inflammatory form of cardiomyopathy distinct from typical dilated or arrhythmogenic right ventricular cardiomyopathy. Circulation.

[10] Kim M.-J., Jung H.O., Kim H., Bae Y., Lee S.Y., Jeon D.S. (2023). 10-year survival outcome after clinically suspected acute myocarditis in adults: A nationwide study in the pre-COVID-19 era. PLoS ONE.

[11] Sinagra G., Anzini M., Pereira N.L., Bussani R., Finocchiaro G., Bartunek J., Merlo M. (2016). Myocarditis in clinical practice. Mayo Clin. Proc..

[12] Bemtgen X., Kaier K., Rilinger J., Rottmann F., Supady A., von Zur Mühlen C., Westermann D., Wengenmayer T., Staudacher D.L. (2024). Myocarditis mortality with and without COVID-19: Insights from a national registry. Clin. Res. Cardiol..

[13] Kwan T.N., Kwan G., Brieger D., Kritharides L., Chow V., Ng A.C.C. (2024). Changing Epidemiology of Myocarditis in Australia: A Population-Based Cohort Study. J. Clin. Med..

[14] Semenzato L., Le Vu S., Botton J., Bertrand M., Jabagi M.-J., Drouin J., Cuenot F., Zores F., Dray-Spira R., Weill A. (2024). Long-Term Prognosis of Patients With Myocarditis Attributed to COVID-19 mRNA Vaccination, SARS-CoV-2 Infection, or Conventional Etiologies. JAMA.

[15] Ng A.C.C., Chung T., Sze Chiang Yong A., Siu Ping Wong H., Chow V., Celermajer D.S., Kritharides L. (2011). Long-term cardiovascular and noncardiovascular mortality of 1023 patients with confirmed acute pulmonary embolism. Circ. Cardiovasc. Qual. Outcomes.

[16] Australian Bureau of Statistics Statistical Area Level 2. https://www.abs.gov.au/statistics/standards/australian-statistical-geography-standard-asgs-edition-3/jul2021-jun2026/main-structure-and-greater-capital-city-statistical-areas/statistical-area-level-2.

[17] Collier J.L., Weiss S.A., Pauken K.E., Sen D.R., Sharpe A.H. (2021). Not-so-opposite ends of the spectrum: CD8+ T cell dysfunction across chronic infection, cancer and autoimmunity. Nat. Immunol..

[18] Martins W.d.A., Schlabendorff E. (2022). Myocarditis in Cancer patients: A review of an emerging problem in Cardio-Oncology. ABC Heart Fail. Cardiomyopathy.

[19] Badawy M., Revzin M.V., Consul N., Soliman M., Ganeshan D.M., Heymann J.C., Gaballah A.H., Rao Korivi B., Morani A.C., Javadi S. (2023). Paraneoplastic syndromes from head to toe: Pathophysiology, imaging features, and workup. Radiographics.

[20] Nie J., Zhou L., Tian W., Liu X., Yang L., Yang X., Zhang Y., Wei S., Wang D.W., Wei J. (2025). Deep insight into cytokine storm: From pathogenesis to treatment. Signal Transduct. Target. Ther..

[21] Clarke A.E., Pooley N., Marjenberg Z., Langham J., Nicholson L., Langham S., Embleton N., Wang X., Desta B., Barut V. (2021). Risk of malignancy in patients with systemic lupus erythematosus: Systematic review and meta-analysis. Semin. Arthritis Rheum..

[22] Kim Y., Seidman J.G., Seidman C.E. (2022). Genetics of cancer therapy-associated cardiotoxicity. J. Mol. Cell Cardiol..

[23] Zhong R., Fang Z., Polychronidis G., Lo C.-H., Knudsen M.D., He M.-M., Wang K., Wang L., Song M. (2022). Immune-mediated diseases associated with cancer risks. JAMA Oncol..

[24] Ammirati E., Cipriani M., Lilliu M., Sormani P., Varrenti M., Raineri C., Petrella D., Garascia A., Pedrotti P., Roghi A. (2017). Survival and left ventricular function changes in fulminant versus nonfulminant acute myocarditis. Circulation.

[25] Fu M., Kontogeorgos S., Thunström E., Zverkova Sandström T., Kroon C., Bollano E., Schaufelberger M., Rosengren A. (2022). Trends in myocarditis incidence, complications and mortality in Sweden from 2000 to 2014. Sci. Rep..

[26] Ghanizada M., Kristensen S.L., Bundgaard H., Rossing K., Sigvardt F., Madelaire C., Gislason G.H., Schou M., Hansen M.L., Gustafsson F. (2021). Long-term prognosis following hospitalization for acute myocarditis–a matched nationwide cohort study. Scand. Cardiovasc. J..

[27] Grün S., Schumm J., Greulich S., Wagner A., Schneider S., Bruder O., Kispert E.-M., Hill S., Ong P., Klingel K. (2012). Long-term follow-up of biopsy-proven viral myocarditis: Predictors of mortality and incomplete recovery. J. Am. Coll. Cardiol..

[28] Younis A., Mulla W., Matetzky S., Masalha E., Afel Y., Fardman A., Goitein O., Arad M., Mazin I., Beigel R. (2020). Sex-based differences in characteristics and in-hospital outcomes among patients with diagnosed acute myocarditis. Am. J. Med..

[29] McNamara D.M., Starling R.C., Cooper L.T., Boehmer J.P., Mather P.J., Janosko K.M., Gorcsan J., Kip K.E., Dec G.W., Investigators I. (2011). Clinical and demographic predictors of outcomes in recent onset dilated cardiomyopathy: Results of the IMAC (Intervention in Myocarditis and Acute Cardiomyopathy)-2 study. J. Am. Coll. Cardiol..

[30] Georgiopoulos G., Figliozzi S., Sanguineti F., Aquaro G.D., di Bella G., Stamatelopoulos K., Chiribiri A., Garot J., Masci P.G., Ismail T.F. (2021). Prognostic impact of late gadolinium enhancement by cardiovascular magnetic resonance in myocarditis: A systematic review and meta-analysis. Circ. Cardiovasc. Imag..

[31] Nedkoff L., Lopez D., Hung J., Knuiman M., Briffa T.G., Murray K., Davis E., Aria S., Robinson K., Beilby J. (2022). Validation of ICD-10-AM coding for myocardial infarction subtype in hospitalisation data. Heart Lung Circ..

[32] Gedeborg R., Holm L., Feltelius N., Sundström A., Eggers K.M., Nurminen M.-L., Grünewald M., Pihlström N., Zethelius B., Ljung R. (2023). Validation of myocarditis diagnoses in the Swedish patient register for analyses of potential adverse reactions to COVID-19 vaccines. Ups. J. Med. Sci..

[33] Zee-Cheng C.S., Tsai C.C., Palmer D.C., Codd J.E., Pennington D.G., Williams G.A. (1984). High incidence of myocarditis by endomyocardial biopsy in patients with idiopathic congestive cardiomyopathy. J. Am. Coll. Cardiol..

[34] Gravanis MB S.N. (1991). Incidence of myocarditis. A 10-year autopsy study from Malmö, Sweden. Arch. Pathol. Lab. Med..

[35] Mieno M.N., Tanaka N., Arai T., Kawahara T., Kuchiba A., Ishikawa S., Sawabe M. (2016). Accuracy of death certificates and assessment of factors for misclassification of underlying cause of death. J. Epidemiol..

[36] Tsampras T., Antonopoulos A., Kasiakogias A., Mika A., Kolovou A., Papadimitriou E., Lazaros G., Tsioufis K., Vlachopoulos C. (2025). Cardiac Magnetic Resonance to Reclassify Diagnosis and Detect Cardiomyopathies in Hospitalized Patients with Acute Presentation. Life.

[37] Shojania K.G., Burton E.C. (2008). The vanishing nonforensic autopsy. N. Engl. J. Med..

