# Supplementary material for: Cause-Specific Mortality in Patients Hospitalized for Myocarditis from 2004 to 2021: A Retrospective Statewide Population-Linkage Study
Source: J Clin Med. 2025 Jun 10;14(12):4089. doi: 10.3390/jcm14124089 (PMC12194032; doi:10.3390/jcm14124089)
Supplement: Supplementary file 1 [file jcm-14-04089-s001.zip › jcm-3664865-supplementary.pdf]

Supplementary materials

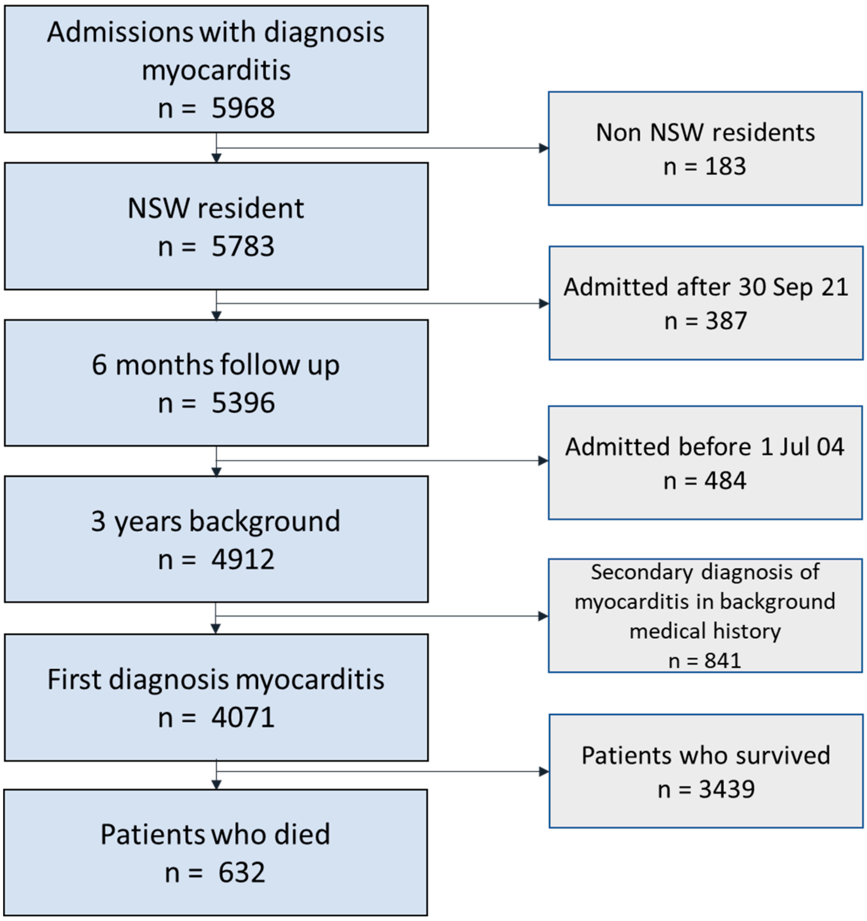

Supplementary Figure S1: Patient selection flowchart

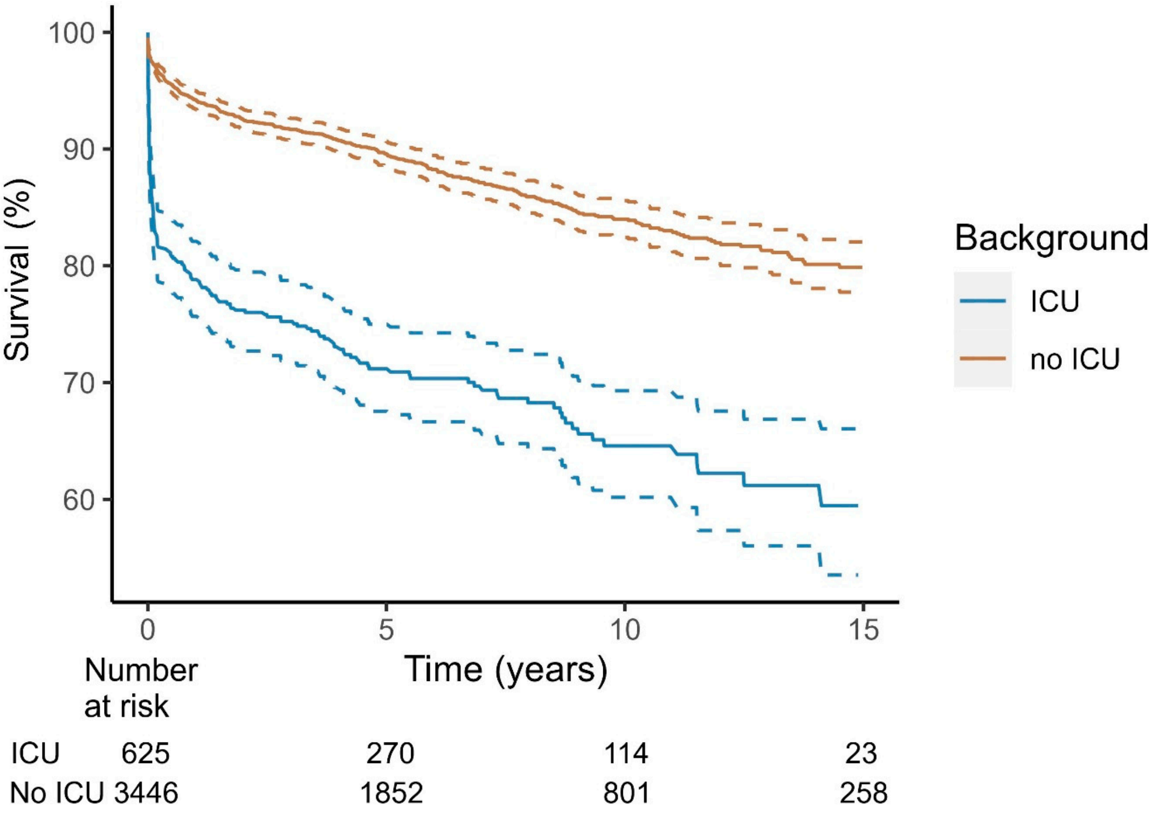

Supplementary Figure S2: Kaplan-Meier plot of survival after myocarditis diagnosis stratified by admission to intensive care unit during index hospitalization

Dotted lines demarcate 95% confidence interval

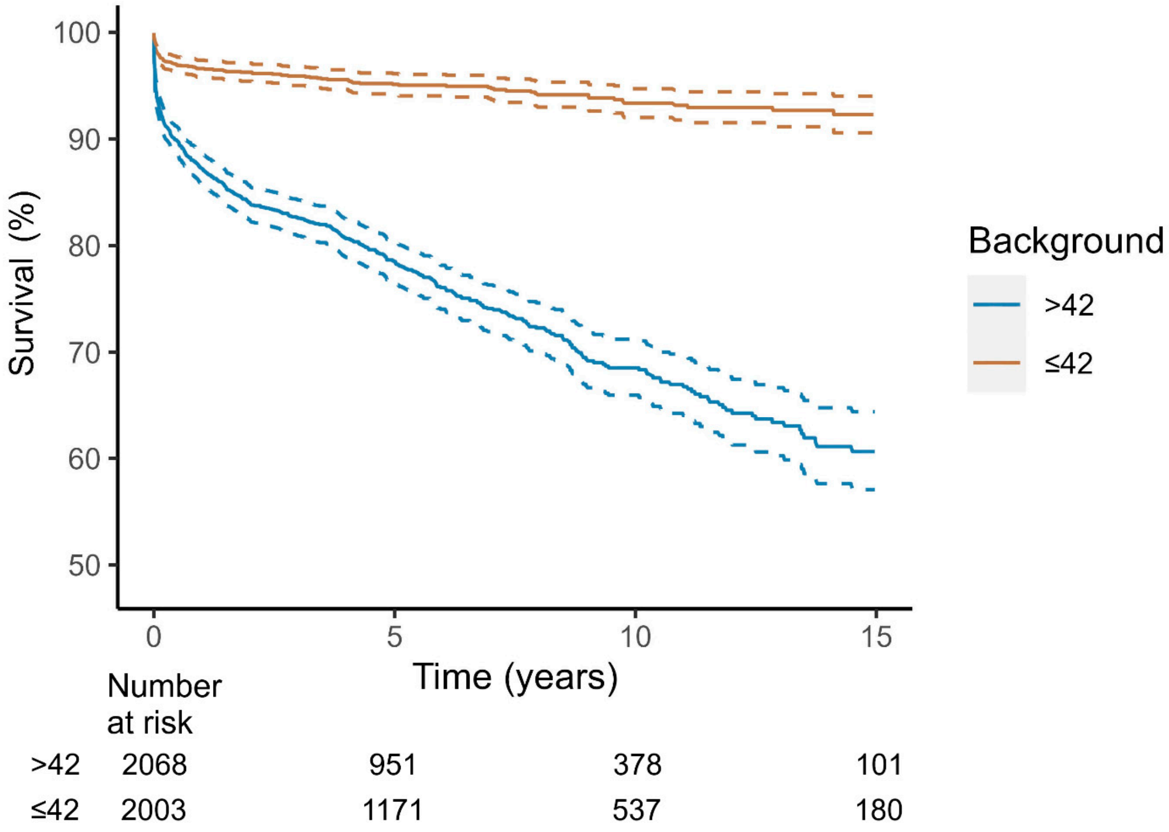

Supplementary Figure S3: Kaplan-Meier plot of survival after myocarditis diagnosis stratified by median age (42 years) at index hospitalization

Dotted lines demarcate 95% confidence interval

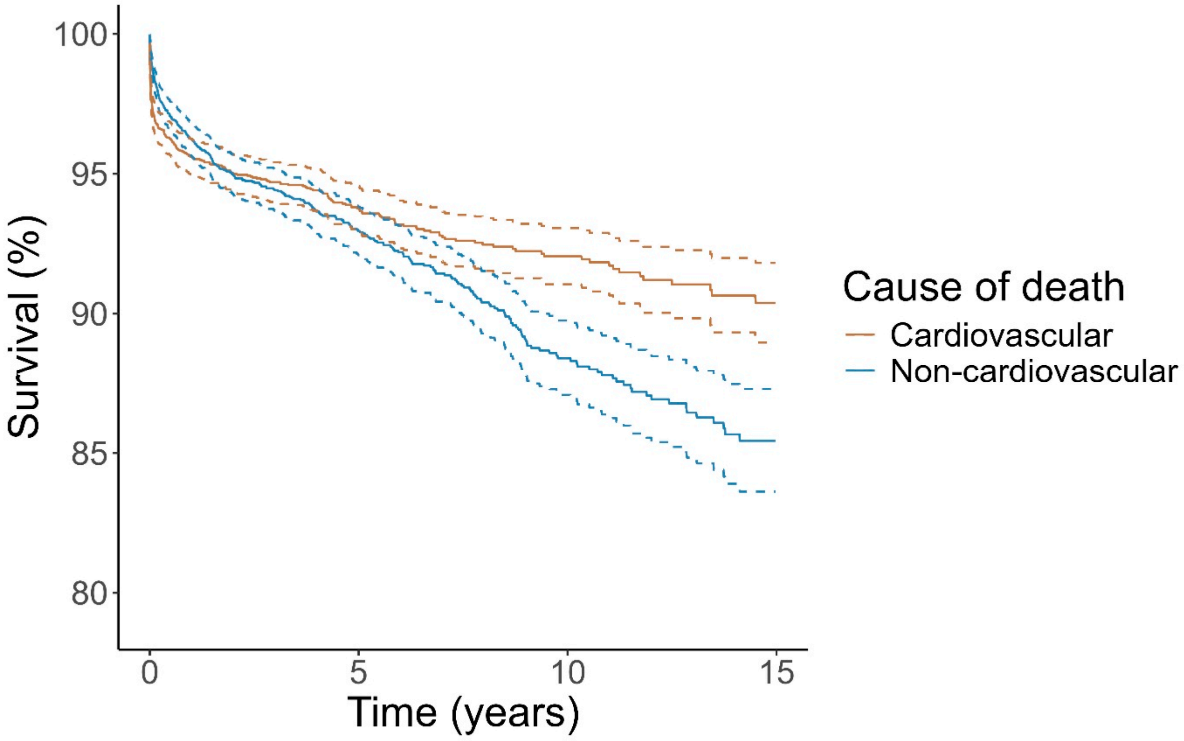

Supplementary Figure S4: Kaplan Meier plot of survival after myocarditis diagnosis according to cardiovascular and non-cardiovascular causes of death

Dotted lines demarcate 95% confidence interval.

Each line represents a separately modelled Kaplan Meier curve.

Supplementary Table S1: The International Classification of Disease (ICD-10AM) and Australian Classification of Health Intervention (ACHI) codes used in the present study

Diagnosis	ICD-10AM or ACHI code
Atrial fibrillation	I48, I48.0, I48.1, I48.2, I48.3, I48.4, I48.9
AIDS	B20, B21, B22, B23, B23.0, B23.8, B24
Chronic kidney disease	N18, N18.3, N18.4, N18.5, N18.9, N19, I12.0, I13.1, U87.1, Z49, Z49.0, Z49.1, Z49.2, Z94.0, Z99.2
Covid	U06.0, U07.1, U07.2, U07.7
CTD	M30, M30.0, M30.1, M30.2, M30.3, M30.8, M31, M31.0, M31.1, M31.2, M31.3, M31.4, M31.5, M31.6, M31.7, M31.8, M31.9, M32, M32.0, M32.1, M32.8, M32.9, M33, M33.0, M33.1, M33.2. M33.9, M34, M34.0, M34.1, M34.2, M34.8, M34.9, M35, M35.0, M35.1, M35.2, M35.3, M35.4, M35.5, M35.6, M35.7, M35.8, M35.9, M36, M36.0, M36.1, M36.2, M36.3, M36.4, M36.8
Dilated cardiomyopathy	I42.0
Dementia	F00, F00.0, F00.1, F00.2, F00.9, F01, F01.0, F01.2, F01.3, F01.8, F01.9, F02, F02.0, F02.1, F02.2, F02.3, FO2.4, FO2.8, F03, F05.1, U79.1, G30, G30.0, G30.1, G30.8, B30.9
Diabetes	E10, E10.0, E10.00, E10.01, E10.02, E10.1, E10.10, E10.11, E10.12, E10.13, E10.14, E10.15, E10.16, E10.2, E10.20, E10.21, E10.22, E10.23, E10.29, E10.3, E10.30, E10.31, E10.32, E10.33, E10.34, E10.35, E10.36, E10.39, E10.4, E10.40, E10.41, E10.42, E10.43, E10.49, E10.5, E10.50, E10.51, E10.52, E10.53, E10.59, E10.6, E10.60, E10.61, E10.62, E10.63, E10.64, E10.65, E10.69, E10.7, E10.70, E10.71, E10.73, E10.8, E10.80, E10.81, E10.9, E10.90, E10.91, E11, E11.0, E11.00, E11.01, E11.02, E11.1, E11.10, E11.11, E11.12, E11.13, E11.14, E11.15, E11.16, E11.2, E11.20, E11.21, E11.22, E11.23, E11.29, E11.3, E11.30, E11.31, E11.32, E11.33, E11.34, E11.35, E11.36, E11.39, E11.4, E11.40, E11.41, E11.42, E11.43, E11.49, E11.5, E11.50, E11.51, E11.52, E11.53, E11.59, E11.6, E11.60, E11.61, E11.62, E11.63, E11.64, E11.65, E11.69, E11.7, E11.70, E11.71, E11.72, E11.73, E11.8, E11.80, E11.81, E11.9, E11.90, E11.91, E12, E12.0, E12.00, E12.01, E12.1, E12.10, E12.11, E12.2, E12.20, E12.21, E12.3, E12.30, E12.31, E12.4, E12.40, E12.41, E12.5, E12.50, E12.51, E12.6, E12.60, E12.61, E12.7, E12.70, E12.71, E12.8, E12.80, E12.81, E12.9, E12.90, E12.91, E13, E13.0, E13.00, E13.01, E13.02, E13.1, E13.10, E13.11, E13.12, E13.13, E13.14, E13.15, E13.16, E13.2, E13.20, E13.21, E13.22, E13.23, E13.29, E13.3, E13.30, E13.31, E13.32, E13.33, E13.34, E13.35, E13.36, E13.39, E13.4, E13.40, E13.41, E13.42, E13.43, E13.49, E13.5, E13.50, E13.51, E13.52, E13.53, E13.59, E13.6, E13.60, E13.61, E13.62, E13.63, E13.64, E13.65, E13.69, E13.7, E13.70, E13.71, E13.72, E13.73, E13.8, E13.80, E13.81, E13.9, E13.90, E13.91, E14, E14.0, E14.00, E14.01, E14.02, E14.1, E14.10, E14.11, E14.12, E14.13, E14.14, E14.15, E14.16, E14.2, E14.20, E14.21, E14.22, E14.23, E14.29, E14.3, E14.30, E14.31, E14.32, E14.33, E14.34, E14.35, E14.36, E14.39, E14.4, E14.40, E14.41, E14.42, E14.43, E14.49, E14.5, E14.50, E14.51, E14.52, E14.53, E14.59, E14.6, E14.60, E14.61, E14.62, E14.63, E14.64, E14.65, E14.69, E14.7, E14.70, E14.71, E14.72, E14.73, E14.8, E14.80, E14.81, E14.9, E14.90, E14.91

Emphysema	J41, J41.0, J41.1, J41.8, J42, J43, J43.0, J43.1, J43.2, J43.8, J43.9, J44, J44.0, J44.1, J44.8, J44.9, J45, J45.0, J45.1, J45.8, J45.9, J46, J47, U83.1, U83.2, U83.3, J98.2, J98.3
Endocarditis	I33, I33.0, I33.9, I38, I39, I39.0, I39.1, I39.2, I39.3, I39.4, I39.8
Heart failure	I11, I11.0, I13.0, I13.1, I13.2, I42, I43, I42, I42.0, I42.1, I42.2, I42.3, I42.4, I42.5, I42.6, I42.7, I42.8, I42.9, I43, I43.0, I43.1, I43.2, I43.8, I50, I50.0, I50.9, I50.1, I13.53, I14.53, I25.5 #NB: does not include U82.2, E10.53, E11.53
Hemiplegia	G80.02, G80.1, G80.2, G81, G81.0, G81.1, G81.9, G82, G82.0, G82.00, G82.01,G82.02, G82.03, G82.04, G82.05, G82.06, G82.1, G82.10, G82.11, G82.12,G82.13, G82.14, G82.15, G82.16, G82.2, G82.20, G82.21, G82.22, G82.23,G82.24, G82.25, G82.26, G82.3, G82.30, G82.31, G82.32, G82.33, G82.34,G82.35, G82.36, G82.4, G82.40, G82.41, G82.42, G82.43, G82.44, G82.45,G82.46, G82.5, G82.51, G82.52, G82.53, G82.54, G82.55, G82.56,U80.5, G11.4, M62.3, M62.30, M62.31, M62.32, M62.33, M62.34, M62.35, M62.36, M62.37, M62.38, M62.39
HIV	B20, B21, B22, B23, B23.0, B23.8, B24, F02.4, O98.7, R75, Z21
Hypertension	I10, I11, I11.0, I11.9, I12, I12.0, I12.9, I13, I13.0, I13.1, I13.2, I13.9, I15, I15.0, I15.1, I15.2, I15.8, I15.9, U82.3
Hyperlipidaemia	E78, E78.0, E78.1, E78.2, E78.3, E78.4, E78.5, E78.6, E78.8, E78.9
Inflammatory bowel disease	K50, K50.0, K50.1, K50.8, K50.9, K51, K51.0, K51.2, K51.3, K51.4,K51.5, K51.8, K51.9, M07.4, M07.40, M07.41, M07.42, M07.43, M07.44,M07.45, M07.46, M07.47, M07.48, M07.49, M07.5, M07.50, M07.51,M07.52, M07.53, M07.54, M07.55, M07.56, M07.57, M07.58, M07.59,M07.6, M07.61, M07.62, M07.63, M07,64, M07.65, M07.66, M07.67,M07.68, M07.69, U84.1, U84.2, M09.1, M09.10, M09.11, M09.12,M09.13, M09.14, M09.15, M09.16, M09.17, M09.18, M09.19, M09.2,M09.20, M09.21, M09.22, M09.23, M09.24, M09.25, M09.26, M09.27,M09.28, M09.29
Ischaemic heart disease	I20, I20.0, I20.1, I20.8, I20.9, I21, I21.0, I21.1, I21.2, I21.3, I21.4, I21.9, I22, I22.0, I22.1, I22.8, I22.9, I23, I23.0, I23.1, I23.2, I23.3, I23.4, I23.5, I23.6, I23.8, I24, I24.0, I24.1, I24.8, I24.9, I25, I25.0, I25.1, I25.10, I25.11, I25.12, I25.13, I25.2, I25.3, I25.4, I25.5, I25.6, I25.8, I25.9
Influenza	J09, J10, J10.0, J10.1, J10.8, J11, J11.0, J11.1, J11.8
Leukaemia	C90.1, C90.10, C90.11, C91, C91.0, C91.00, C91.01, C91.1, C91.10, C91.11, C91.2, C91.20, C91.21, C91.3, C91.30, C91.31, C91.4, C91.40, C91.41, C91.5, C91.50, C91.51, C91.6, C91.60, C91.61, C91.7, C91.70, C91.71, C91.8, C91.80, C91.81, C91.9, C91.90, C91.91, C92, C92.0, C92.00, C92.01, C92.1, C92.10, C92.11, C92.2, C92.20, C92.21, C92.4, C92.40, C92.41, C92.5, C92.50, C92.51, C92.6, C92.60, C92.61, C92.7, C92.70, C92.71, C92.8, C92.80, C92.81, C92.9, C92.90, C92.91, C93, C93.0, C93.00, C93.01, C93.1, C93.10, C93.11, C93.2, C93.20, C93.21, C93.3, C93.30, C93.31, C93.7, C93.70, C93.71, C93.9, C93.90, C93.91, C94, C94.0, C94.00, C94.01, C94.2, C94.20, C94.21, C94.3, C94.30, C94.31, C94.7, C94.70, C94.71, C95, C95.0, C95.00, C95.01, C95.1, C95.10, C95.11, C95.2, C95.20, C95.21, C95.7, C95.70, C95.71, C95.9, C95.90, C95.91, D47.5, Z85.6, M9733/3, M9742/3, M980, M9800/3, M9801/3, M9802/3, M9803/3, M9804/3, M9805/3, M9806/3, M9807/3, M9808/3,

	M9809/3, M981-M983, M9811/3, M9812/3, M9813/3, M9814/3, M9815/3, M9816/3, M9817/3, M9818/3, M9820/3, M9821/3, M9822/3, M9823/3, M9824/3, M9825/3, M9826/3, M9827/3, M982-M983, M9830/3, M9831/1, M9832/3, M9833/3, M9834/3, M9835/3, M9836/3, M9837/3, M9840/3, M984-M993, M9850/3, M9860/3, M9861/3, M9862/3, M9863/3, M9864/3, M9865/3, M9866/3, M9867/3, M9868/3, M9869/3, M9870/3, M9871/3, M9872/3, M9873/3, M9874/3, M9875/3, M9876/3, M9880/3, M9890/3, M9891/3, M9892/3, M9893/3, M9894/3, M9895/3, M9896/3, M9897/3, M9898/3, M9900/3, M9910/3, M9911/3, M994, M9940/3, M9945/3, M9946/3, M9948/3, M9963/3, M9964/3
Liver disease	K70, K70.0, K70.1, K70.2, K70.3, K70.4, K70.9, K71, K71.0, K71.1, K71.2, K71.3, K71.4, K71.5, K71.6, K71.7, K71.8, K71.9, K72, K72.0, K72.1, K72.9, K73, K73.0, K73.1, K73.2, K73.8, K73.9, K74, K74.0, K74.1, K74.2, K74.3, K74.4, K74.5, K74.6, K75,K75.0, K75.1, K75.2, K75.3, K75.4, K75.8, K75.9, K76, K76.0, K76.1, K76.2,K76.3, K76.4, K76.5, K76.6, K76.7, K76.8, K76.9,B18, B18.0, B18.1, B18.2, B18.8, B18.9, Z94.4,I85, I85.0, I85.9, I86.4, I98.2, I98.20, I98.21, I98.3
Moderate to severe liver disease	K70.4, K71.1, K72, K72.0, K72.1, K72.9, K76.7, I85, I85.0, I85.9, I86.4, I98.2, I98.20, I98.21, I98.3
Lymphoma	C81, C81.0, C81.1, C81.2, C81.3, C81.4, C81.7, C81.9, C82, C82.0, C82.1, C82.2, C82.3, C82.4, C82.5, C82.6, C82.7, C82.9, C83, C83.0, C83.1, C83.2, C83.3, C83.4, C83.5, C83.6, C83.7, C83.8, C83.9, C84, C84.2, C84.3, C84.4, C84.5, C84.6, C84.7, C84.8, C84.9, C85, C85.1, C85.2, C85.7, C85.9, C86, C86.0, C86.1, C86.2, C86.3, C86.4, C86.5, C88.4, C88.40, C88.41, C91.5, C91.50, C91.51, C96.3, M959, M9590/3, M9591/3, M9595/3, M9596/3, M9597/3, M959-M972, M9650/3, M9651/3, M9653/3, M9654/3, M9655/3, M9659/3, M965-M966, M9663/3, M9664/3, M9665/3, M9667/3, M9670/3, M9671/3, M9672/3, M9673/3, M9674/3, M9675/3, M9676/3, M9677/3, M9678/3, M9679/3, M967-M969, M967-M972, M9680/3, M9681/3, M9682/3, M9683/3, M9684/3, M9685/3, M9686/3, M9687/3, M9688/3, M9689/3, M969, M9690/3, M9691/3, M9692/3, M9693/3, M9694/3, M9695/3, M9696/3, M9697/3, M9698/3, M9699/3, M9702/3, M9703/3, M9704/3, M9705/3, M9706/3, M9707/3, M9708/3, M9709/3, M970-M971, M971, M9711/3, M9712/3, M9713/3, M9714/3, M9716/3, M9717/3, M9719/3, M972, M9723/3, M9725/3, M9726/3, M9727/3, M9728/3, M9729/3, M9735/3, M9737/3, M9738/3, C88.4, C88.40, C88.41
Myocarditis	I01.2, I09.0, I40, I40.0, I40.1, I40.8, I40.9, I41, I41.0, I41.1, I41.2, I41.8, I51.4
Myositis	M33, M33.0, M33.1, M33.2, M33.9, M60, M60.0, M60.00, M60.01, M60.02, M60.03, M60.04, M60.05, M60.07, M60.07, M60.08, M60.09, M60.1, M60.10, M60.11, M60.12, M60.13, M60.14, M60.15, M60.16, M60.17, M60.18, M60.19, M60.8, M60.80, M60.81, M60.82, M60.83, M60.84, M60.85, M60.86, M60.87, M60.88, M60.89, M60.9, M60.90, M60.91, M60.92, M60.93, M60.94, M60.95, M60.96, M06.97, M60.98, M60.99, M63, M63.0, M63.00, M63.01, M63.02, M63.03, M63.04, M63.05, M63.07, M63.07, M63.08, M63.09, M63.1,M63.10, M63.11, M63.12, M63.13, M63.14, M63.15, M63.16, M63.17, M63.18, M63.19, M63.2, M63.20, M63.21, M63.22, M63.23, M63.24,

	M63.25, M63.26, M63.27, M63.28, M63.29, M63.3, M63.30, M63.31, M63.32, M63.33, M63.34, M63.35, M63.36, M63.37, M63.38, M63.39
Pericarditis	I01.0, I09.2, I30, I30.1, I30.8, I30.9, I31.0, I31.1, I32, I32.0, I32.1, I32.8
Psoriatic arthritis	M07.0, M07.00, M07.04, M07.07, M07.09, M07.1, M07.10, M07.12, M07.13, M07.14, M07.15, M07.16, M07.17, M07.18, M07.19, M07.2, M07.3, M07.30, M07.31, M07.32, M07.33, M07.34, M07.35, M0.36, M07.37, M07.38, M07.39
Peripheral vascular disease	W09.01, E09.02, E09.5, E09.51, E09.52, E10.51, E10.52, E11.51,E11.52, E13.51, E13.52, E14.51, E14.52, I70, I70.0, I70.1, I70.2,I70.20, I70.21, I70.22, I70.23, I70.24, I70.8, I70.9, I71, I71.0,I71.00, I71.01, I71.02, I71.03, I71.1, I71.2, I71.3, I71.4, I71.5,I71.6, I71.8, I71.9, I72, I72.0 I72.1, I72.2, I72.3, I72.4, I72.5,I72.6, I72.8, I72.9, I73, I73.0, I73.1, I73.8, I73.9, I74, I74.0,I74.1, I74.2, I74.3, I74.4, I74.5, I74.8, I74.9, I77, I77.0, I77.1,I77.2, I77.3, I77.4, I77.5, I77.6, I77.8, I77.9, I78, I78.0, I78.1,I78.8, I78.9, I79, I79.0, I79.1, I79.2, I79.8
Rheumatoid arthritis	M05, M05.0, M05.00, M05.01, M05.02, M05.03, M05.04, M05.05, M05.06, M05.07, M05.08, M05.09, M05.1, M05.10, M05.11, M05.12, M05.13, M05.14, M05.15, M05.16, M05.17, M05.18, M05.19, M05.2, M05.20, M05.21, M05.22, M05.23, M05.24, M05.25, M05.26, M05.27, M05.28, M05.29, M05.3, M05.30, M05.31, M05.32, M05.33, M05.34, M05.35, M05.36, M05.37, M05.38, M05.39, M05.8, M05.80, M05.81, M05.82, M05.83, M05.84, M05.85, M05.86, M05.87, M05.88, M05.89, M05.9, M05.90, M05.91, M05.92, M05.93, M05.94, M05.95, M05.96, M05.97, M05.98, M05.99, M06, M06.0, M06.00, M06.01, M06.02, M06.03, M06.04, M06.05, M06.06, M06.07, M06.08, M06.09, M06.2, M06.20, M06.21, M06.22, M06.23, M06.24, M06.25, M06.26, M06.27, M06.28, M06.29, M06.3, M06.30, M06.31, M06.32, M06.33, M06.34, M06.35, M06.36, M06.37, M06.38, M06.39, M06.8, M06.80, M06.81, M06.82, M06.83, M06.84, M06.85, M06.86, M06.87, M06.88, M06.89, M06.9, M06.90, M06.91, M06.92, M06.93, M06.94, M06.95, M06.96, M06.97, M06.98, M06.99, U86.1
Sarcoidosis	D86, D86.0, D86.1, D86.2, D86.3, D86.8, D86.9, G53.2, M63.3, M63.30, M63.31, M63.32, M63.33, M63.34, M63.35, M63.36, M63.37, M63.38, M63.39))#NB: there is no code for cardiac sarcoidosis of any form
Systemic lupus erythematosus	L93, L93.0, L93.1, L93.2, M32, M32.0, M32.1, M32.8, M32.9, U86.3
Smoking	Z58.7, F17, F17.0, F17.2, F17.3, F17.4, F17.6, F17.7, F17.8, F17.9, T65.2, Z71.6, Z72.0, Z86.43
Stroke	G46, G46.0, G46.1, G46.2, G46.3, G46.4, G46.5, G46.6, G46.7, G46.8, I63, I63.0, I63.1, I63.2, I63.3, I63.4, I63.5, I63.6, I63.8, I63.9, I69.3, I60, I60.0, I60.1, I60.2, I60.3, I60.4, I60.5, I60.6, I60.7, I60.8, I60.9, I61, I61.0, I61.1, I61.2, I61.3, I61.4, I61.5, I61.6, I61.8, I61.9, I62, I62.0, I62.1, I62.9, I64, I69.0, I69.1, I69.2, I64, I69.4
Syncope	R55
Transient ischaemic attack	G45, G45.0, G45.1, G45.2, G45.3, G45.4, G45.8, G45.9
Ventricular arrhythmia	I47.0, I47.2, I49.0
Venous thromboembolism	I26, I26.0, I26.9, I80, I80.0, I80.1, I80.2, I80.3, I80.8, I80.9, I81, I82, I82.0, I82.1, I82.2, I82.3, I82.8, I82.9

Autoimmune disease	D86, D86.0, D86.1, D86.2, D86.3, D86.8, D86.9, G53.2, L93, L93.0, L93.1, L93.2, MO6, MO6, MO7, MO8, MO9, M30, M31, M32, M33, M34, M35, M36, M45, M46, M63.3, M63.30, M63.31, M63.32, M63.33, M63.34, M63.35, M63.36, M63.37, M63.38, M63.39, U86.3
Malignancy	ICD-10 codes beginning with C
Respiratory disease	ICD-10 codes beginning with J
Digestive disease	ICD-10 codes beginning with K
Chest MRI	90901-04
Defibrillator insertion	38393-00
Heart transplantation	90205-00, 90205-01
Hip fracture	47528-01, 49318-00, 49319-00
Myocardial biopsy	38275-00, 38418-03

Supplementary Table S2: Changing pattern of in-hospital cause-specific mortality after diagnosis of myocarditis over time

Cause of death	Quartile 1: 2004-2011	Quartile 2: 2011-2015	Quartile 3: 2015-2018	Quartile 4: 2018-2021	p-value
Cardiovascular	39 (59.1%)	31 (68.9%)	24 (63.2%)	16 (48.5%)	0.001
Heart failure	1 (1.5%)	10 (22.2%)	7 (18.4%)	4 (12.1%)	0.568
Myocarditis	18 (27.3%)	16 (35.6%)	15 (39.5%)	11 (33.3%)	0.205
Myocardial infarction	15 (22.7%)	4 (8.9%)	0 (0%)	0 (0%)	<0.001
Arrhythmia	3 (4.5%)	0 (0%)	1 (2.6%)	0 (0%)	0.115
Stroke	0 (0%)	1 (2.2%)	1 (2.6%)	1 (3%)	0.450
Pulmonary embolism	2 (3%)	0 (0%)	0 (0%)	0 (0%)	1
Noncardiovascular	24 (36.4%)	13 (28.9%)	14 (36.8%)	16 (48.5%)	0.210
Infection	13 (19.7%)	9 (20%)	8 (21.1%)	6 (18.2%)	0.104
Malignancy	5 (7.6%)	0 (0%)	1 (2.6%)	5 (15.2%)	0.893
Other non-cardiac death	5 (7.6%)	2 (4.4%)	4 (10.5%)	3 (9.1%)	0.633
Pulmonary disease	1 (1.5%)	2 (4.4%)	1 (2.6%)	2 (6.1%)	0.716
Unknown cause of death	3 (4.5%)	1 (2.2%)	0 (0%)	1 (3%)	0.183
Total	129 (100%)	89 (100%)	76 (100%)	65 (100%)	<0.001
Total mortality for each quartile cohort	66/1018 (6.5%)	45/1018 (4.4%)	38/1017 (3.7%)	33/1018 (3.2%)	-

4 quantiles divided number of patients evenly.
p-value calculated by Poisson regression, treating time of diagnosis of myocarditis as an ordinal variable of 4 possible values. The 4 quantiles were separated by the precise dates: 3-July-2004, 22-April-2011, 12-November-2015, 26-December-2018 and 28-September-2021.

Supplementary Table S3: Changing pattern of 30-day cause-specific mortality after diagnosis of myocarditis over time

Cause of death	Quartile 1: 2004-2011	Quartile 2: 2011-2015	Quartile 3: 2015-2018	Quartile 4: 2018-2021	p-value
Cardiovascular	38 (61.3%)	35 (77.8%)	23 (63.9%)	19 (59.4%)	0.001
Heart failure	1 (1.6%)	10 (22.2%)	7 (19.4%)	3 (9.4%)	0.568
Myocarditis	17 (27.4%)	20 (44.4%)	13 (36.1%)	13 (40.6%)	0.205
Myocardial infarction	15 (24.2%)	4 (8.9%)	0 (0%)	1 (3.1%)	<0.001
Arrhythmia	3 (4.8%)	0 (0%)	1 (2.8%)	0 (0%)	0.115
Stroke	0 (0%)	1 (2.2%)	2 (5.6%)	2 (6.2%)	0.45
Pulmonary embolism	2 (3.2%)	0 (0%)	0 (0%)	0 (0%)	1
Noncardiovascular	21 (33.9%)	9 (20%)	13 (36.1%)	13 (40.6%)	0.21
Infection	11 (17.7%)	7 (15.6%)	8 (22.2%)	4 (12.5%)	0.104
Malignancy	4 (6.5%)	0 (0%)	1 (2.8%)	5 (15.6%)	0.893
Other non-cardiac death	5 (8.1%)	0 (0%)	3 (8.3%)	3 (9.4%)	0.633
Pulmonary disease	1 (1.6%)	2 (4.4%)	1 (2.8%)	1 (3.1%)	0.716
Unknown cause of death	3 (4.8%)	1 (2.2%)	0 (0%)	0 (0%)	0.183
Total	121 (100%)	89 (100%)	72 (100%)	64 (100%)	-
Total mortality for each quartile cohort	121/1018 (11.9%)	89/1018 (8.7%)	72/1017 (7.1%)	64/1018 (6.3%)	<0.001

4 quantiles divided number of patients evenly.

p-value calculated by Poisson regression, treating time of diagnosis of myocarditis as an ordinal variable of 4 possible values. The 4 quantiles were separated by the precise dates: 3-July-2004, 22-April-2011, 12-November-2015, 26-December-2018 and 28-September-2021.

Supplementary Table S4: Cause-specific mortality in patients with Covid-19 myocarditis

**Cause of death**	**Quartile 1: 2004-2011**
Cardiovascular	8 (38%)
Heart failure	2 (10%)
Myocarditis	4 (19%)
Myocardial infarction	0
Arrhythmia	1 (5%)
Stroke	1 (5%)
Pulmonary embolism	0
Noncardiovascular	13 (62%)
Infection	6 (29%)
Malignancy	5 (24%)
Other non-cardiac death	1 (5%)
Pulmonary disease	1 (5%)
Unknown cause of death	0
Total	21 (100%)

Covid-19 myocarditis defined as myocarditis within 30 days of admission with Covid-19

Supplementary Table S5: Univariable predictors of mortality following myocarditis admission

Variable	n	Hazard ratio (95% CI)	p-value	C-index
Age (per 10 years)	4071	1.69 (1.61-1.76)	<0.001	0.75
Sex (male)	2690	0.66 (0.57-0.78)	<0.001	0.54
ICU	625	2.8 (2.36-3.32)	<0.001	0.6
City hospital	3007	0.96 (0.8-1.14)	0.61	0.51
Rural hospital	235	0.9 (0.63-1.27)	0.544	0.5
ASGS[a]	4071	1.01 (0.89-1.15)	0.879	0.51
Background chronic kidney disease	187	5.46 (4.38-6.81)	<0.001	0.56
Background diabetes	423	3.31 (2.75-3.98)	<0.001	0.57
Background ischaemic heart disease	1110	2.46 (2.1-2.88)	<0.001	0.59
Background hypertension	1090	3.49 (2.98-4.08)	<0.001	0.63
Background hyperlipidaemia	320	2.16 (1.75-2.68)	<0.001	0.53
Background chronic obstructive pulmonary disease	485	2.49 (2.05-3.02)	<0.001	0.56
Background liver disease	315	3.38 (2.75-4.17)	<0.001	0.56
Background autoimmune disease	229	2.02 (1.55-2.62)	<0.001	0.52
Background malignancy	321	6.25 (5.2-7.5)	<0.001	0.61
Complicated by atrial fibrillation	239	2.7 (2.15-3.4)	<0.001	0.54
Complicated by ventricular arrhythmia	584	2.49 (2.09-2.97)	<0.001	0.58
Complicated by heart failure	162	2.34 (1.73-3.14)	<0.001	0.53
Complicated by stroke	29	5.5 (3.35-9.05)	<0.001	0.51
Charlson comorbidity index	4071	1.42 (1.39-1.45)	<0.001	0.81
Covid-19 myocarditis*	281	1.13 (0.64-2.01)	0.669	0.51

a. Unadjusted Cox regression analysis performed

[a]ASGS: Australian Statistical Geography Standard, where higher number associated with more remoteness (i.e. hazard ratio below 1 indicates lower death in more remote centres)

*Analysis restricted to cases after 25 January 2020

Supplementary Table S6: Predictors of mortality following myocarditis admission in multivariable model in cohort restricted to after Covid-19 pandemic[a]

Variable	n	Adjusted hazard ratio (95% CI)	p-value
Covid-19 myocarditis	281	0.9 (0.47-1.73)	0.757
Age (per 10 years)	663	1.76 (1.36-2.26)	<0.001
Sex (male)	417	1.62 (0.85-3.08)	0.144
ICU	90	1.83 (0.87-3.86)	0.11
ASGS[b]	663	1.26 (0.72-2.18)	0.421
Background chronic kidney disease	41	1.56 (0.7-3.46)	0.273
Background diabetes	82	2.06 (1.05-4.02)	0.034
Background ischaemic heart disease	181	1.03 (0.54-1.99)	0.92
Background hypertension	226	1.77 (0.78-4.02)	0.17
Background hyperlipidaemia	38	0.72 (0.27-1.96)	0.525
Background chronic obstructive pulmonary disease	126	2.45 (1.23-4.87)	0.01
Background liver disease	71	2.55 (1.1-5.89)	0.029
Background autoimmune disease	51	0.66 (0.25-1.69)	0.383
Background malignancy	69	2.6 (1.35-5.02)	0.004
Complicated by atrial fibrillation	32	2.78 (1.11-6.94)	0.029
Complicated by heart failure	75	1.09 (0.46-2.6)	0.845
Complicated by ventricular arrhythmia	29	2.12 (0.64-6.99)	0.217
Complicated by stroke	5	9.67 (2.01-46.45)	0.005

a. Analysis restricted to cases after 25 January 2020

b. ASGS: Australian Statistical Geography Standard, where higher number associated with more remoteness (i.e. hazard ratio below 1 indicates lower death in more remote centers)

b. C-index of model: 0.91
